# Religious Affiliation as a Predictor of Involuntary Psychiatric Admission: A Brazilian 1-Year Follow-Up Study

**DOI:** 10.3389/fpubh.2014.00102

**Published:** 2014-08-11

**Authors:** Caio Borba Casella, Alexandre Andradade Loch

**Affiliations:** ^1^Department and Institute of Psychiatry, School of Medicine, Universidade de São Paulo, São Paulo, Brazil

**Keywords:** involuntary hospitalization, religion, psychosis, bipolar, re-hospitalization

## Abstract

**Objective:** The aim of this study was to analyze factors associated with the legal status at psychiatric admission of individuals with psychosis or bipolar disorder in a Latin-American cultural setting.

**Methods:** Prospective observational study was conducted in São Paulo, Brazil. We analyzed 169 individuals with bipolar or psychotic disorder in need of hospitalization. Sociodemographic data, data on the psychiatric disorder, information about the hospital stay, and data at time of discharge were collected. Their families were also contacted by telephone and interviews were conducted at 1, 2, 6, and 12 months post-discharge as a follow-up.

**Results:** Eighty-eight patients (52%) had a voluntary admission and 81 (48%) had an involuntary admission (IA). The average length of admission was similar in both groups (17.4 vs. 17.3 days, *p* = 0.22). It was significantly more common for IA patients to be admitted because of other-directed aggressiveness (47.7 vs. 65.4%, *p* = 0.02). The percentage of individuals that needed physical restraint during hospital stay among IA patients was also significantly higher (11.4 vs. 25.9%, *p* = 0.01). Having any religious affiliations was significantly related to an IA status as well (OR = 4–6.48).

**Conclusion:** Our results suggest that cultural factors related to religious affiliations might play an important role in determining psychiatric hospitalization legal status. Religion might possibly influence someone’s judgment and insight about his/her psychiatric disorder. This study restates the importance of dealing with the subject of religion with patients.

## Introduction

An involuntary admission (IA) is sometimes found necessary in the course of the treatment of a patient with psychiatric disorders (1). Among the commonest reasons considered for an IA are: (a) a severe mental disorder, (b) danger to self and others and (c) (urgent) need for treatment, although there are some variations in each country legislation (2). However, despite the evidence supporting that IA is sometimes indispensable, hospitalizing someone against his/her will is still a controversial topic.

It is often pointed out that an IA and coercive practices could be considered an infringement of individual freedom (1) and might have negative consequences in the treatment. It might create a feeling of exclusion from participation in the treatment process (3) and greater levels of dissatisfaction with it (4). Katsakou and Priebe (5) found that a substantial number of patients following an IA do not feel that their admission was justified (10–47%) or beneficial (6–33%) for their treatment, even though most of them do show significant clinical symptom and functional improvement. Patient may also become disempowered and stigmatized, with reduced self-esteem and quality of life (6). Coercive practices might create an aversion to the medical treatment (7) and consequently reduce the probability of the patient seeking care. A review study (4) found that the outcome of an involuntary hospital admission, in terms of length of stay, readmission risk and risk of involuntary readmission, was at least equal, if not worse, to the outcome of a voluntary admission (VA).

As such, international research aimed to determine which factors were related to an IA. As yet associations were found for male gender (8–12), single status (8, 9, 11), a diagnosis of psychosis (9–12), higher aggressive behavior at admission (8, 11), severe psychopathology (8, 11), and low level of social functioning (8), although results are not always consistent. Surprisingly, religion is usually neglected in these studies, even though it is often recognized as an important aspect regarding mental health (13). Religion might be a source of coping for the patient (14) but, at the same time, some religious practices might discourage the seeking and acceptance of medical care (15), regarding the psychiatric symptoms only as a spiritual manifestation (16).

Also, despite the growing effort of research on this topic, most of these studies are still restricted to North American and European countries (4), such that information in other contexts is lacking. Different to other countries, for instance, a great percentage of Brazilian population is religious – about 92% declares some religious affiliation (17), with a significant proportion of them being evangelic. Previous studies have suggested that evangelic are more resistant to seek formal psychiatric help (18). Therefore, religion might play an important role in influencing the population that seeks medical help voluntarily in our setting.

The main objective of our study was to assess factors related to an IA, including religious affiliation. Secondarily, we analyzed whether psychiatric admission legal status had an effect on 1-year re-hospitalization rates. To accomplish that, we studied a representative sample of public mental health system users with the diagnosis of psychosis and bipolar disorder of São Paulo, the most populous Brazilian metropolis.

## Materials and Methods

### Background

The present study was conducted in São Paulo. It is the largest Brazilian metropolis and the sixth most populous city in the world, with an estimated population of 11 million inhabitants (19). As in most parts of the world, a major mental health care reform has been carried out over the last 20 years in the country (20, 21): a national public health system was created (Sistema Unico de Saude, SUS); the number of psychiatric hospital beds has been drastically reduced; and many Centers for Psychosocial Care (Centro de Atenção Psicossocial, CAPS), community outpatients services, were created (21, 22). CAPS are supposed to be intensive outpatient services, where individuals in more need of assistance can attend the service more than once a week. Each CAPS has a multidisciplinary team consisting of psychiatrists, psychologists, nurses, social workers, and occupational therapists. Besides hospital beds and CAPS, there are the Basic Health Units (Unidade Básica de Saúde, UBS), community services, many with psychiatrists, where patients are seen on a monthly basis. At the time of the study, there were 5 stand-alone psychiatric hospitals, 10 general hospitals with psychiatric beds, 43 CAPS, and 546 UBS operating in the city (23). According to studies published in the last decade, though there are critical issues regarding mental healthcare resources organization in Brazil (22), figures are similar to that of other developed countries where a psychiatric reform was conducted (24–26).

Regarding psychiatric admission legislation, likewise in other countries, there are three modes of hospitalization: voluntary, involuntary, and compulsory (27). Whereas compulsory admission is only possible when there is a court order for it, IA can be conducted if there is: (1) risk of other-directed aggression, (2) risk of self-aggression, (3) risk of aggression to the public order, (4) risk of social exposure, or (5) severe inability to self-care. Each involuntary hospitalization must be informed to the Public Ministry within 72 h of admission. A designated commission will judge them if the IA is valid/legal and intervene if necessary. In theory, IA can be converted to VA and vice-versa during the course of the hospital stay, but in practice this seldom occurs.

### Sampling and procedure

Subjects were recruited from the “Philippe Pinel Psychiatric Hospital,” one of five public stand-alone psychiatric hospitals of São Paulo. This facility has 36 acute psychiatric beds for males and 12 for females, and is responsible for almost 10% of the hospitalizations of individuals with psychosis and bipolar disorder (ICD-10 F2 and F31/F30) of the metropolis (26).

Patients are only admitted to the facility through emergency units located throughout the city. Individuals are referred from these units to the hospital on an as-needed basis; thus, inpatients originate from all regions of the metropolitan area. Also, specifically at this hospital, all psychiatric diagnoses are accepted for hospitalization, the only exception being subjects with substance use disorder as the primary diagnosis; these individuals are admitted elsewhere. Hence, individuals at this hospital are considered representative of the city’s bipolar and psychotic disorder patients who required hospitalization in the public health system.

Sample comprised adult subjects consecutively discharged from the hospital from May to August 2009. At each month of the study, patients and their families were consecutively asked at the time of their discharge whether they were willing to participate in the study. After providing a description of the study to the participants, written informed consent was obtained. If a number between 40 and 50 participants was reached for the month, the recruiting was suspended and reinitiated at the beginning of the following month. After discharge, an appointment was made at the CAPS nearest to the patient’s home, as a rule, no more than 1 month after leaving the hospital.

Of the total discharges in this 4-month period (*n* = 271), 216 (79.7%) were consecutively invited to participate in the study. Of these, 176 (81.5%) agreed to collaborate, signed in an informed consent form, and could be contacted at least once after discharge.

The sample had an array of different diagnoses; in order to obtain a more homogenous sample, only individuals with the diagnosis of psychosis or bipolar disorder (ICD-10 F2 or F31/F30) were considered for analysis; individuals with substance use disorder (*n* = 1, 0.6%), unipolar depression (*n* = 2, 1.1%), mental retardation (*n* = 1, 0.6%), and organic mental disorder (*n* = 3, 1.7%) as the primary diagnosis were not considered. This resulted in a final sample of 169 individuals.

Following, a social worker contacted the patient’s family by telephone at four different time points; 1, 2, 6, and 12 months after discharge from the index hospitalization. The social worker tried to reach every family at the specific time points several times. If contact was not made in the month designated for the team to call the family, it was considered a refusal. From the 169 families, 145 (86%) were contacted in the first month, 136 (81%) in the second month, 134 (79%) at month 6, and 108 (64%) at month 12.

Our study has been approved by the Ethics Committee of the City of São Paulo.

### Instrument and measures

The instrument was a specific questionnaire designed for the study. The first part was filled out by the main investigator with data from the patient’s hospital file. Sociodemographic data, data on the psychiatric disorder, information about the hospital stay, and the hospital discharge were collected.

The second part was filled out by the social worker by telephone interviews. It consisted of six parts; (1) individual’s general behavior; (2) attendance to his/her outpatient consultations, and if not, reason why; (3) medication adherence; (4) in this part, the interviewer asked whether the subject was re-hospitalized since index hospitalization (re-hospitalization was also considered if the patient was readmitted elsewhere than hospital where the study was conducted), including reason why the individual was re-hospitalized; (5) information about drug use; (6) questions about the family’s opinion on psychiatric hospitalization (“Do you agree with brief hospitalization?”; “Do you wish your relative had stayed a longer time at the hospital?”; “Do you agree with permanent hospitalization of the mentally ill?”).

Point-estimates of re-hospitalization in the various interview schedules (1, 2, 6, and 12 months) were used. That is, for these variables, only data obtained at those specific time points were considered. A further variable was created representing the survival to re-admission (“re-hospitalization survival”), considering data obtained from all time points. More detailed description of methods can be found elsewhere (28).

Outcome variables did not show statistically significant differences between individuals with psychosis and individuals with bipolar disorder, so sample was analyzed as a whole and not split according to diagnosis.

### Statistical analysis

Participants and non-participants were compared: Mann–Whitney tests were conducted for the continuous variables, age, antipsychotics dose at discharge, and duration of hospital stay (all of them had significant *p*-values in the Shapiro–Wilk test, indicating a non-normal distribution); for categorical variables sex, diagnosis of entry and of discharge, number of previous hospitalizations and time since the illness began Chi-square tests were used. Afterwards, descriptive statistics compared individuals with voluntary vs. IA, using the same tests according to continuous or categorical variables.

Backward stepwise logistic regression was conducted; admission voluntariness was set as the dependent variable, and variables significantly different between involuntarily and voluntarily admitted patients were used as predictors. This procedure was used to fit fewer parameters into the regression model, to result in a model with more precise estimates. Next, admission voluntariness was used to conduct two distinct Kaplan–Meyer survival curves. Log rank was used to estimate statistical difference between the two curves.

All statistical analyses were conducted with SPSS 18.0 (PASW) for Windows; two-tailed tests and level of significance (*p*) of 0.05 were used.

## Results

The 169 participants did not statistically differ from non-participants (*n* = 47) regarding the variables mentioned in the Section “Materials and Methods.” From the total sample, 88 (52%) had a VA and 81 (48%) had an IA. The vast majority of the sample was male (VA = 78.4%, IA = 80.2%), and single (VA = 88.2%, IA = 86.4%), with mean age of 38.8 (VA) and 36.2 (IA) years (Table 1). These demographic variables did not differ statistically between groups.

**Table 1:** **Sample characteristics according to admission status (*n* = 169)**.

	Voluntary	Involuntary	*p*
	(*n* = 88)	(*n* = 81)	
	% (*n*)	% (*n*)	
**SOCIODEMOGRAPHICAL DATA**			
Sex (male)	78.4 (69)	80.2 (65)	0.77
Civil state (single)	88.2 (75)	86.4 (70)	0.69
Age (years)	38.8*	36.2*	0.97
Religion
Catholic	**28.6 (20)**	**36.4 (24)**	**0.00**
Evangelic	**25.7 (18)**	**42.4 (28)**	
No religion	**40.0 (28)**	**13.6 (9)**	
Receiving disability payments	32.5 (25)	36.9 (24)	0.58
**FACTORS RELATED TO THE ILLNESS/PRIOR TO ADMISSION**			
Number of previous admissions (11 or more)	22.2 (18)	31.5 (23)	0.59
Time since first episode (11 or more years)	61.9 (52)	62.0 (49)	0.84
Correctly using medication before admission	43.5 (37)	41.7 (30)	0.81
Drug use before admission	26.9 (21)	31.9 (22)	0.51
Family history of psychiatric disorder	43.0 (37)	50.0 (40)	0.37
**FACTORS RELATED TO ADMISSION**			
First admission	1.1 (1)	1.3 (1)	0.95
Diagnosis at entry (psychosis)	63.9 (46)	68.5 (37)	0.59
Cause of admission
Other-directed aggressiveness	**47.7 (42)**	**65.4 (53)**	**0.02**
Self-aggressiveness	15.9 (14)	11.1 (9)	0.36
Suicide risk	5.7 (5)	4.9 (4)	0.83
Outpatient treatment failure	13.6 (12)	6.2 (5)	0.11
Psychomotor agitation	40.9 (36)	39.5 (32)	0.85
Needed physical restraint during hospital stay	**11.4 (10)**	**25.9 (21)**	**0.01**
Received family visitation during hospital stay	61.4 (54)	70.4 (57)	0.22
Length of admission (days)	17.4*	17.3*	0.94
Antipsychotics at discharge
Atypical	22.7 (20)	25.9 (21)	0.63
Typical	88.6 (78)	87.7 (71)	0.84
Depot	19.3 (17)	14.8 (12)	0.44
Antipsychotic dose at discharge (in chlorpromazine equivalents)	8.20*	8.12*	0.74
**POST-DISCHARGE DATA**			
Family does not agree with brief hospitalization	78.7 (66)	67.1 (53)	0.17
Family agrees with permanent hospitalization	43.4 (36)	43.0 (34)	0.97
Family would like their relative to have stayed longer at the hospital	83.7 (72)	75.9 (60)	0.21
Is not attending outpatient consultation after discharge	40.9 (36)	50.0 (40)	0.24
Is not taking medication after discharge	26.4 (23)	35.0 (28)	0.23

*Measured as a mean. Bold font indicates p < 0.05.

In both groups, over 60% of patients had 11 or more years since the first psychiatric episode and only slightly over 40% declared correct use of medication before admission. The majority of patients received a diagnosis of psychosis (F2x, according to ICD 10) (VA = 63.9%, IA = 68.5%). The average length of admission was similar in both groups (around 17 days) and the majority of patients received typical antipsychotics at discharge (VA = 8.20, IA = 8.12, in chlorpromazine equivalents). In general, patients’ families preferred a longer hospital stay, with around 43% of them agreeing with permanent hospitalization. Treatment attendance after discharge was overall low (40.9% in the VA group and 50% in the IA not attending their outpatient consultations), as well as medication compliance (26.4% in the VA group and 35% in the IA not taking their medication). All the above measures were not statistically different between groups.

Three variables were statistically different between the VA and the IA group; it was significantly more common for IA patients to be admitted because of other-directed aggressiveness (65.4 vs. 47.7%, *p* = 0.02). The percentage of individuals that needed physical restraint during hospital stay among IA patients was also significantly higher (25.9 vs. 11.4%, *p* = 0.01). And a significantly higher percentage of people declared not having a religion in the VA group (40.0%), with fewer patients belonging to the catholic or evangelic religions in this group (*p* < 0.01).

Logistic regression analysis was conducted with factors that yielded significant associations in the descriptive analysis. Table 2 showed that, in this model, having any religious affiliations was related to IA status (OR = 3.42–6.10). Also other-directed aggressiveness as cause of admission was significantly associated with the legal status at admission (OR = 2.70, *p* = 0.02).

**Table 2:** **Results from logistic regression (backwards WALD)**.

Variable	OR 95% CI	*p*
Religion
No religion	Ref.	
Catholic	3.42(1.3–9.3)	0.02
Evangelic	5.25(1.9–14.7)	<0.01
Others	6.10(1.2–30.1)	0.03
Other-directed aggressiveness as cause of admission	2.70(1.2–5.6)	0.02

Kaplan–Meier survival analysis showed that individuals from both IA and VA groups had similar re-hospitalization rates throughout the study period, with around 50% of them requiring readmission by the end of the 12-month study period (Log rank *p* = 0.73) (data not shown).

## Discussion

To the extent of the authors’ knowledge, this is the first study analyzing factors related to legal status at psychiatric admission at Latin America. We compared patients with VA and IA to a psychiatric hospital in Brazil. Our sample was most constituted of male and single patients, with a diagnosis of psychosis at admission. The majority of patients had 11 or more years since the diagnosis and most of them had at least 11 hospital admissions previously. They declared an overall low treatment adherence before admission. Both groups had a similar mean length of hospital stay. Other-directed aggressiveness at admission predicted an IA, as well as having a religion.

Regarding the characteristics of our sample, it is not surprising that the majority of our sample was single, as seen in previous studies (8, 29, 30), since patients with more severe psychopathology might have difficulties establishing a relationship. Furthermore, most of them had multiple previous admissions and were non-adherent before our index hospitalization. Our pre-admission adherence rate was lower than what was seen in previous studies for patients with schizophrenia (50–60%), but slightly higher than that for bipolar patients (as low as 35%) (31). As expected, medication non-compliance increases the risk of rehospitalization (32, 33), so a great part of our participants might probably be referred as “revolving door” patients, that is, patients with a high frequency of hospital admissions and discharges (34). Further on the clinical aspects of the sample, over 60% of them received a diagnosis of psychosis. Previous studies have found an association with such diagnosis and an IA (9, 10). We might not have seen such association in our study because our sample was restricted to patients with diagnosis of psychosis or bipolar disorders.

Despite being the commonest cause of admission in both VA and IA groups, other-directed aggressiveness was one of the main factors that predicted an IA. Correspondingly, our IA patients needed significantly more physical restraint during hospital stay. This finding is similar to previous literature findings (8) that associated high scores of an aggressiveness scale to a higher risk of involuntary hospitalization. In a European study on coercive measures (35), aggression against others was the commonest reason for prescribing them. The involuntary legal status on admission itself has been associated with disruptive behavior during hospital stay (11) and has been considered a predictor of the necessity of coercive measures (36, 37).

An interesting finding was that having any religious affiliation was one of the most important factors associated with an involuntary status at admission, yielding the highest ORs. In contrast to our finding, a review by Bonelli and Koenig (38) reported mostly a positive relationship between some form of religious involvement and better mental health. It is known that many people with schizophrenia use religion to cope with their mental illness (39, 40). It might help them reduce distress, anxiety, and non-adaptive behaviors associated with psychotic symptoms and may help them increase their social support (13, 40, 41). Religion seems also to protect against suicide attempts (14, 39) and participation in group religious practices might help increase adherence to treatment (15).

However, religion may affect treatment negatively as well. Some people may refuse psychiatric care due to their religious beliefs, considering spiritual recovery exclusively (15). Over 30% of bipolar patients, in a New Zealand study (42), declared some incompatibility between their faith and the treatment proposed by mental health professionals. In a Brazilian study on public beliefs in psychiatric treatment (18), Evangelicals showed less preference for various formal treatment options for case vignettes of depression and schizophrenia, including seeking psychiatric help, than other groups. This would be in line with our findings, showing that perhaps individuals in need of psychiatric care would be treated against their will, or against their religious beliefs, by means of an IA.

Additionally, Evangelical (protestant) religion has had a great development in our country in recent decades, with a growing number of followers (18). In the 2010 census, people with this religious affiliation corresponded to about 22% of the population of our country (17) and, correspondingly, they constitute a significant part of our sample. In another study, Moss et al. (43) reported that catholic and protestant patients with psychotic symptoms took a longer time to seek psychiatric treatment than patients with no religious affiliation. With a similar finding, an Indian study (44) reported that around 45% of psychiatric patients sought religious healers prior to psychiatric consultation, delaying medical care. That resistance in seeking mental health care may have a significant impact on the outcome of these patients. In our case, it was more associated with an IA, but it can also yield a longer duration of untreated psychosis, for instance, being associated with a worse prognosis, with higher rates of refractoriness and cognitive decline (43).

Besides this resistance to seek treatment, there could also be a bias regarding our sample: conservative religious groups tend to seek medical help only for those patients with severe psychopathology, retaining in the religious community, with their own ideas of care, patients with less symptoms (45). A previous study by Loch et al. (46) has demonstrated the existence of a continuum of psychotic symptoms in the population of our setting, with some patients presenting these symptoms, but not seeking help and remaining functional in the community. Part of this group of patients might be those religious. Thus, the sample of patients with a religious affiliation that got to our hospital would be constituted, as a mean, of more complex cases and a higher proportion of them would require an IA. What speaks against it is the fact that they did not differ, however, regarding the mean antipsychotic dosage at discharge, an indirect measure of severity.

One could expect that patients who needed an IA would be in a worse clinical state, with more severe symptoms and more resistant to adhere with treatment. Submitted to an IA (47), they would require a longer stay at the hospital and probably relapse more (11). In our study, however, both groups had similar lengths of admission, post-discharge adherence, and re-hospitalization rates, reinforcing previously published works (4, 5, 48–50).

Concerning the limitations of our study, we did not use a structured instrument to evaluate severity of symptoms either at admission or discharge and severity of disorder has been considered a significant association of commitment status in hospital studies (11). However, we did use some indirect measures of severity, such as the need for physical restraint during admission and the antipsychotic dosage at discharge, and there was no statistical difference in these parameters after multivariate analysis. We also restricted our sample to patients with a psychosis and bipolar diagnosis. Psychiatric patients with other diagnosis might have different outcomes regarding the legal status at admission, but we decided to restrict the diagnosis in order to establish a more homogeneous sample.

We did not evaluate subjective perception of coercion or patients’ opinion toward IA, which are important factors that should be considered, since not only it might influence the outcome of the treatment, but also other relevant aspects, such as the patients suffering with the stigma of mental illness (51). However, IA did not interfere with treatment compliance after discharge, so it might not have influenced so negatively their perception of the treatment.

## Conclusion

Our findings suggest that, in our Latin-American sociocultural setting, having a religious affiliation plays an important role in psychiatric admission legal status. Religion is sometimes neglected in psychiatric evaluation and this study restates the relevance of this subject. Religious patients might be more resistant to seeking medical help and complying with the treatment. As a result, they might receive higher rates of coercive measures. When they seek mental health services, they might be at a more severe clinical state, with a worse perception of their mental health, and, thus, require higher rates of IA.

To better understand the reasons of our findings, more studies should be conducted in different cultural settings, which might have significant differences in religiosity. Since the influence of culture in psychiatry is well-documented [e.g., culture-bound syndromes (52)], it would be important to clarify the relationship of psychiatry and religion itself in a determined cultural context.

## Author Contributions

Caio Borba Casella contributed to the interpretation of the data of this present work, as well as drafting it. Alexandre Andradade Loch contributed to the conception of this work, as well as acquisition, analysis, and interpretation of data and revised it. Caio Borba Casella and Alexandre Andradade Loch both agree to be accountable for all aspects of this present work.

## Conflict of Interest Statement

The authors declare that the research was conducted in the absence of any commercial or financial relationships that could be construed as a potential conflict of interest.
